# The conservative approach for infantile gastric volvulus

**DOI:** 10.1007/s00383-025-06007-9

**Published:** 2025-05-05

**Authors:** Irem Inanc, Sadettin Yildiz, Umit Nusret Basaran, Dincer Avlan

**Affiliations:** 1 Department of Pediatric Surgery, Trakya University Faculty of Medicine, 22030 Edirne, Turkey; 2 Department of Pediatric Surgery, Division of Pediatric Urology, Trakya University Faculty of Medicine, 22030 Edirne, Turkey

**Keywords:** Organoaxial, Mesenteroaxial, Vomiting, Infant, Conservative

## Abstract

**Objectives:**

This study evaluates the outcomes of conservative management of chronic gastric volvulus in a series of neonates, with a focus on diagnostic and therapeutic approaches.

**Methods:**

A retrospective review was conducted on 13 patients diagnosed with chronic gastric volvulus between 2015 and 2024. The clinical and imaging data were analyzed, including age at diagnosis, sex, presenting symptoms, treatment type, and follow-up outcomes. Diagnosis was confirmed with contrast-enhanced upper gastrointestinal radiography. Conservative treatment involved gradual enteral feeding via orogastric tube, specific positional strategies, and total parenteral nutrition.

**Results:**

Of the 13 patients (7 female, 6 male; mean age: 39.23 days), 12 were successfully managed conservatively, with only one requiring surgical gastropexy. The most common presenting symptom was non-bilious vomiting. The mean follow-up was 30.61 months (95% CI: 18.37–42.86 months) revealed all patients achieved weight above the 10th percentile. Conservative management showed a 90.9% success rate, significantly higher than previously reported rates.

**Conclusion:**

Chronic gastric volvulus, often misdiagnosed as GERD, requires clinical suspicion and contrast-enhanced imaging for accurate diagnosis. Conservative management is effective, reducing the need for surgical intervention when standardized protocols are applied.

## Introductıon

Gastric volvulus is a rare but significant cause of non-bilious vomiting, defined as a rotation of the stomach exceeding 180 degrees along its axis [1]. The stomach is normally fixed by the gastrophrenic, gastrohepatic, gastrosplenic, and gastrocystic ligaments. The absence or structural abnormalities of one or more of these ligaments may lead to gastric volvulus [2, 3]. In mesenteroaxial volvulus, the rotation occurs along the short axis of the stomach, whereas organoaxial volvulus involves the long axis [7]. First reported by Berti in 1886, Borchardt in 1904 described the classical triad of “retching without vomiting, sudden epigastric pain, and distension with the inability to pass a nasogastric tube” [4]. Gastric volvulus is classified as acute or chronic for diagnosis and treatment purposes and by the axis of rotation as organoaxial, mesenteroaxial, or combined type. Acute gastric volvulus is a life-threatening type requiring immediate intervention, often presenting as acute gastric outlet obstruction with non-bilious vomiting. Chronic volvulus, in contrast, typically involves progressive symptoms like failure to thrive and recurrent vomiting [1, 5]. While acute gastric volvulus often coexists with diaphragmatic anomalies, associated anomalies are rare in the chronic form [4, 6].

Gastric volvulus is considered rare in children; however, a significant proportion of cases occur in children under the age of 1 year [8]. Particularly in neonates, chronic gastric volvulus can be easily overlooked due to the common yet nonspecific symptom of recurrent non-bilious vomiting. Some experts propose that chronic gastric volvulus is not as rare as it is underdiagnosed [9]. The treatment for acute gastric volvulus is surgical, aiming to correct the underlying cause and perform gastropexy. However, the success rate of conservative treatment in chronic volvulus has been reported in the literature to vary between 40% and 100%. [3, 9, 13]. Although surgical intervention has an overall high success rate, it is associated with increased morbidity and should be reserved for refractory cases [1]. Given the immaturity and increased laxity of suspensory ligaments in neonates, conservative treatment is considered a more suitable option in this specific age group, with higher success rates compared to surgical intervention [10].

This study aims to evaluate the outcomes of our case series, the majority of whom were diagnosed with chronic gastric volvulus during the neonatal period and to review the diagnostic and therapeutic approaches in light of the existing literature.

## Materials and methods

The medical records of patients diagnosed with gastric volvulus through clinical and imaging studies, consulted from our hospital’s neonatal intensive care unit and pediatric emergency department between 2015 and 2024 due to non-bilious vomiting and feeding intolerance, were retrospectively reviewed. Data on age at diagnosis, sex, presenting symptoms, type of treatment, follow-up duration, and percentile information at the final follow-up were recorded. All patients suspected of having gastric volvulus underwent a contrast-enhanced upper gastrointestinal (GI) study, which was used to confirm the diagnosis in every case and the types of gastric volvulus were documented.

The conservative treatment protocol was defined as following diagnosis, enteral feeding was initiated immediately without an NPO period, gradually increasing enteral feeding via an orogastric tube with breast milk or formula while the patient received total parenteral nutrition. Feeding was performed with the patient positioned at a 45-degree angle, followed by placement on the right side to facilitate gastric emptying and restore the stomach to its normal anatomical position. None of the patients were treated with prokinetic agents or proton pump inhibitors. Failure of conservative treatment was defined as an inability to increase feed volume over 7 days, absence of weight gain or occurrence of weight loss, gastric residuals exceeding the feed volume post-feeding, or persistent vomiting.

## Results

**Fig. 1:**
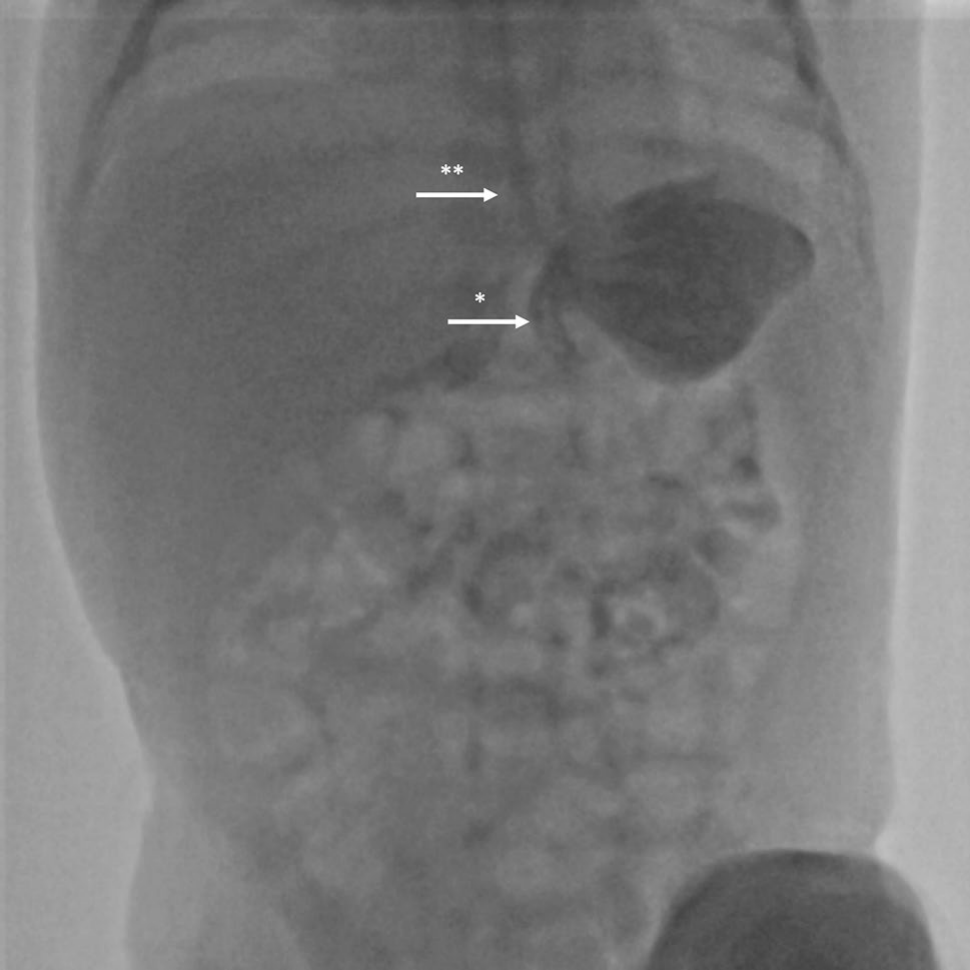
Mesenteroaxial gastric volvulus; *represents pylorus and **represents esophagus, note the superior positioning of the pylorus

**Fig. 2:**
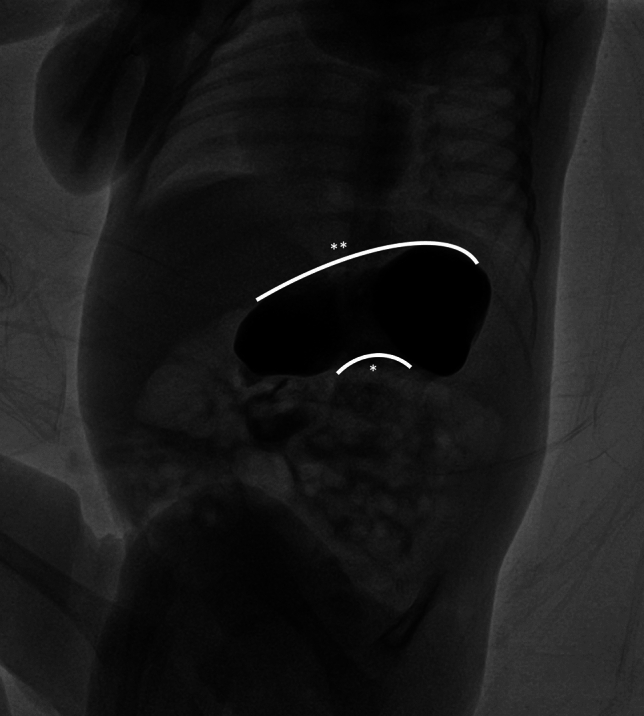
Organoaxial gastric volvulus; *represents lesser curvature, **represents greater curvature, Note the superior positioning of the greater curvature

The demographic and clinical data of the patients are summarized in Table 1. A total of 13 patients were diagnosed with chronic gastric volvulus and followed during the study period. Seven of the patients were female and six were male, with a mean age at diagnosis of 39.23 days (range: 4–210 days). Five patients were diagnosed with mesenteroaxial gastric volvulus (Fig. 1), while eight had organoaxial volvulus (Fig. 2).

**Table 1:** Demographic and clinical data of the patients

	Sex*	Age at diagnosis (day)	Indication for hospitalization	Type of gastric volvulus**	Additional anomaly	Treatment	Follow-up period(month)	Last percentile***
Case 1	M	45	Vomiting, weight loss	MA	–	Conservative	42	10–25
Case 2	F	4	Vomiting, weight loss	MA	–	Conservative	36	25
Case 3	F	90	Vomiting, weight loss	OA	Cystic fibrosis, syndactyly	Conservative	36	25–50
Case 4	M	30	Vomiting, weight loss	MA	–	Conservative	30	75
Case 5	F	210	Vomiting, weight loss, malnutrition	OA	–	Conservative	30	25
Case 6	M	30	Prematurity	OA	–	Conservative	30	10–25
Case 7	M	5	Prematurity	OA	–	Conservative	24	75
Case 8	F	30	Prematurity	MA	–	Conservative	24	50–75
Case 9	F	7	Prematurity	OA	–	Conservative	18	50
Case 10	F	10	Prematurity	OA	–	Conservative	12	50
Case 11	M	30	Hyperbilirubinemia	OA	–	Surgical (open gastropexy)	90	75–97
Case 12	M	12	Vomiting	MA	–	Conservative	13	25–50
Case 13	F	7	Vomiting, weight loss	OA	–	Conservative	13	25–50

*F Female, *M* Male

**MA Mesenteroaxial, *OA* Organoaxial

***The percentile from the most recent application

One patient required surgical intervention who was initially managed conservatively but showed persistent vomiting and significant feeding intolerance during the whole conservative treatment period, prompting the decision for surgery. Open gastropexy was performed. However, the patient developed adhesive intestinal obstruction and required reoperation at postoperative day 28. The patient recovered well following the second surgery, with no further complications or recurrence during follow-up. The remaining 12 patients were treated conservatively, and none subsequently required surgical intervention. The most common reason for neonatal intensive care unit admission was prematurity, while vomiting was the most frequent cause of inpatient admission. An associated anomaly was present in only one patient, and vomiting was a symptom in all cases.

The mean follow-up duration was 30.61 months (range: 12–90 months). At the final follow-up, all patients had achieved a weight above the 10th percentile (Table 1). Figures 1 and 2 show radiographic images from the UGIs.

## Discussion

In the infant age group, chronic gastric volvulus is an under-recognized and underdiagnosed cause of feeding intolerance and non-bilious vomiting [5]. The patients are often initially misdiagnosed and treated as gastroesophageal reflux disease (GERD). Cribbs et al. reported that the incidence of chronic gastric volvulus is higher in studies outside the United States. This was attributed to the routine use of contrast-enhanced UGI in Europe, Asia, and Africa for patients with recurrent vomiting and feeding intolerance, whereas similar cases in the United States are primarily treated for GERD [3]. This observation aligns with the notion that chronic gastric volvulus in infants is not necessarily rare but rather underdiagnosed.

Non-specific symptoms such as feeding intolerance, non-bilious vomiting, and failure to thrive often lead to a presumptive diagnosis of GERD. However, as observed in our series, contrast-enhanced UGI plays a crucial role in diagnosing chronic gastric volvulus. While upper GI studies are essential for confirming gastric volvulus, their diagnostic role must be interpreted in context. A key diagnostic challenge is that, unlike acute volvulus, where nasogastric tube insertion is typically impossible, chronic volvulus can spontaneously resolve and recur, allowing nasogastric tube placement and further complicating the clinical picture. However, this study does not include a direct comparison of GERD-diagnosed cases later confirmed with Upper GI, which remains a limitation.

Recurrent vomiting episodes in gastric volvulus can result in failure to thrive and prolonged hospital stays, particularly in cases with delayed diagnosis [1, 8]. Early recognition and treatment of gastric volvulus in neonates are essential to reduce intensive care unit stays and prevent malnutrition. In our series, symptoms emerged within the first month of life in more than half of the patients, with vomiting-induced weight loss necessitating neonatal intensive care unit admission.

The treatment of acute gastric volvulus generally involves correction of the underlying anomaly and gastropexy, either via laparoscopic or open surgery [2, 6, 11, 12]. Similarly, in cases of chronic gastric volvulus with failed conservative treatment, gastropexy is performed [13], as was the case in one patient in our series. Conservative management for chronic gastric volvulus has been described in the literature, emphasizing positional maneuvers during feeding to restore the stomach to its normal anatomical position. Although large case series on chronic gastric volvulus are limited, Cribbs et al. reported a 40% success rate for conservative treatment [3]. In contrast, Da Costa et al.’s systematic review reported a success rate of 28.9% for overall and 71.43% for chronic gastric volvulus [13]. In the series by Al-Salem et al. [9], which included 34 patients with chronic gastric volvulus, conservative treatment was attempted in 11 patients and success was achieved in all cases. The variability in success rates appears to be primarily due to the lack of large series in the literature and the selective application of conservative treatment to specific patients.

In our series, the success rate for conservative management was 90.9%. This high rate may be attributed to early diagnosis, differentiation from GERD, standardization of conservative treatment protocols, avoidance of premature surgical intervention, and the absence of significant associated anomalies that could worsen the patients’ general condition.

The primary limitations of this study include the small sample size and the limited number of surgically treated patients, preventing a comparative analysis of treatment modalities. Although our study demonstrates a high success rate for conservative treatment, the lack of a direct comparison with surgical intervention remains a limitation. Future prospective studies comparing conservative and surgical outcomes are needed to further support our findings.

During follow-up, no patients experienced recurrence of gastric volvulus, and none required delayed surgical intervention. However, given the limited sample size and follow-up duration, long-term complications beyond our study period remain unknown. This is acknowledged as a limitation of our findings.

In conclusion, vomiting is a common, nonspecific symptom in the infant age group and can be associated with various conditions. Chronic gastric volvulus may be easily overlooked without clinical suspicion, leading to prolonged symptom duration, weight loss, and frequent hospital admissions if untreated. Contrast-enhanced UGI plays a critical role in diagnosis. This condition responds well to conservative treatment, and surgical intervention should not be hastily pursued.

## Data Availability

No datasets were generated or analysed during the current study.
